# Influence of Surface Features for Increased Heat Dissipation on Tool Wear

**DOI:** 10.3390/ma11050664

**Published:** 2018-04-25

**Authors:** Nageswaran Tamil Alagan, Tomas Beno, Philipp Hoier, Uta Klement, Anders Wretland

**Affiliations:** 1 Department of Engineering Science, University West, SE-461 32 Trollhättan, Sweden; tomas.beno@hv.se; 2 Department of Industrial and Materials Science, Chalmers University of Technology, SE-412 96 Gothenburg, Sweden; hoierp@chalmers.se (P.H.); uta.klement@chalmers.se (U.K.); 3 GKN Aerospace Engine Systems AB, SE-461 81 Trollhättan, Sweden; anders.wretland@gknaerospace.com

**Keywords:** Alloy 718, carbide insert, high-pressure coolant, machining, textured inserts, tool-chip contact area

## Abstract

The critical problems faced during the machining process of heat resistant superalloys, (HRSA), is the concentration of heat in the cutting zone and the difficulty in dissipating it. The concentrated heat in the cutting zone has a negative influence on the tool life and surface quality of the machined surface, which in turn, contributes to higher manufacturing costs. This paper investigates improved heat dissipation from the cutting zone on the tool wear through surface features on the cutting tools. Firstly, the objective was to increase the available surface area in high temperature regions of the cutting tool. Secondly, multiple surface features were fabricated for the purpose of acting as channels in the rake face to create better access for the coolant to the proximity of the cutting edge. The purpose was thereby to improve the cooling of the cutting edge itself, which exhibits the highest temperature during machining. These modified inserts were experimentally investigated in face turning of Alloy 718 with high-pressure coolant. Overall results exhibited that surface featured inserts decreased flank wear, abrasion of the flank face, cutting edge deterioration and crater wear probably due to better heat dissipation from the cutting zone.

## 1. Introduction

Alloy 718 is a nickel base alloy with high mechanical and thermal strength in combination with outstanding weldability and creep resistance property at elevated temperature. This makes it a natural choice when producing components for hot sections in jet engines. When it comes to machining, Alloy 718 acts as a thermal insulator which increases the steady-state temperature on the cutting edge [1]. Concentrated heat in the proximity of the cutting edge leads to an increase of the tool wear thus reducing the service life of the cutting tool.

In spite of today’s machining technology and cooling strategies, it is still a problem to dissipate enough heat from the cutting zones during the machining of Alloy 718 and other HRSA materials. To improve the heat dissipation from the cutting zone, a potential solution introduced by Pigott and Colwell in 1952 [2], was to focus the coolant jet to the cutting edge at a high-pressure. This led to a 7–8 fold increase of the tool life [2]. Since then, high-pressure coolant technology has helped the metal cutting industry to advance with respect to improved tool life and surface quality, as well as high productivity. The positive effect of high-pressure coolant jets when cutting Alloy 718 has previously been studied both experimentally [3,4] and numerically [5].

In the early 2000’s, there have been several studies regarding the improvement of tool life by surface texturing, mainly focused on the tribological behavior [6,7,8]. Only little focus was put on textures influencing the heat dissipation rate. Some examples of tribological investigations were on the textures and its influence on wear, friction coefficient, cutting forces and tool life. Results indicated prolonged lifetime and reduced friction and forces [6,7,8]. During the last years, the investigations have been directed towards using textures in combination with high-pressure coolant to improve the heat transfer rate for improving tool life. Tamil Alagan et al. have published works on surface featured inserts with high-pressure coolant for improved heat dissipation from the cutting zone [9,10,11]. Fang and Obikawa [12], investigated on five types of textures on the flank face in combination with high-pressure coolant. A pressure of 13 MPa was used on the flank face during machining Alloy 718. The results showed surface texture away from the cutting edge enhanced the heat transfer from the tool to the coolant. Temperature measurement by use of thermocouple and Energy-dispersive X-ray spectroscopy, (EDS), analysis showed that the textured tool had improved cooling performance [12]. This paper explains the design criteria of different generations of inserts and provides results from the investigation of the tool wear mechanism and tool-chip contact area in correlation to the improved heat dissipation of surface featured cutting tools.

## 2. Surface Features on the Regular Insert

### 2.1. Regular Insert

**Figure 1:**
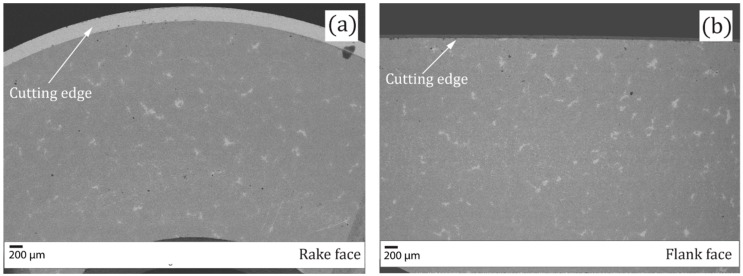
SEM micrograph of regular insert (**a**) Rake face; (**b**) Flank face.

As reference, a commercially available round shaped insert (ISO nomenclature: RCMX 12 04 00 H13A) was used (see Table 1). The present grade consists of tungsten carbide (WC), grains within a matrix of Cobalt (Co) as binder. Scanning electron microscope (SEM) micrograph of rake and flank faces are shown Figure 1.

### 2.2. Surface Features and Design of Generation I Insert

**Figure 2:**
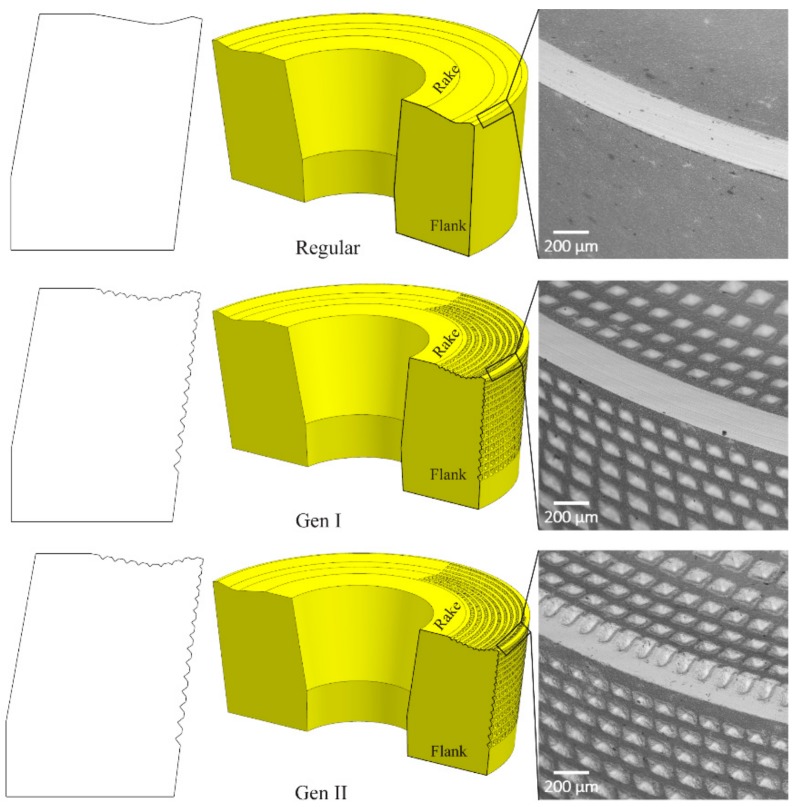
Regular, Gen I and Gen II inserts shows the sectional view and SEM micrographs with the magnified cutting edges.

The Gen I insert was a reengineering of the regular insert (see Figure 2) with the purpose to increase the surface area in regions of high temperature. During the design process, the criterion was to choose a geometry that could yield the largest surface area. Test calculations were performed based on removing a constant volume of 48 mm^3^ for three different geometries; also the corresponding surface area was calculated and is given in Table 2. For instance, a square pyramid of base edge, a, of 4 mm and a height, h, of 9 mm results in in a removed volume of 48 mm^3^. This led to an increase in surface area of 73.76 mm^2^; see Table 2 S.no 1.

The square pyramid geometry has led to an increase of approx. 5.6 times in surface area. From the surface area analysis, a square pyramid geometry has the possibility to create the largest surface area compared to other geometries. Hence, a square pyramid pattern was fabricated by nano-second pulse fiber laser ablation. The pattern was applied at a sector of 90 degrees on the rake and the flank face of the regular insert, see Figure 2.

On the rake face, there are nine rows; the first row of the texture pattern is located at a distance of 0.2 mm from the cutting edge so as not to weaken the cutting edge. The base edge length, *a*, of the first row was 0.086 mm and the height, *h*, was 0.043 mm. For the remaining rows, the base edge length was increased by approximately 10%.

On the flank face there are 14 rows, with the first row being located at a distance of 0.1 mm from the cutting edge. The last row is then placed at a distance of 3.7 mm while the overall thickness of the insert is 4.73 mm. The base edge length, *a*, of the first row was 0.099 mm and the height, *h*, was 0.0518 mm. For the rest of the rows, the base edge length was increased by approximately 10%.

The depth of the indents was increased with the distance from the cutting edge to avoid jeopardizing the structural integrity of the insert. According to Table 2 and computer-aided design (CAD), calculations, the patterns on a sector of 90 degrees of the regular insert resulted in a 12% increase of the surface area.

### 2.3. Design of Generation II Insert

**Figure 3:**
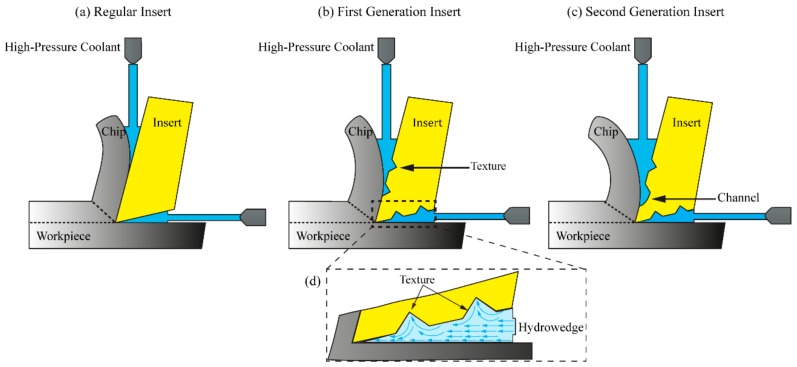
(**a**–**c**) Illustration of the interaction between the different inserts with coolant and chip, and the formation of hydro-wedges on the rake and flank face [10]; (**d**) magnified illustration of coolant-surface feature interaction on the flank face.

The surface features on the rake face close to the cutting edge of Gen I insert were modified based on the theoretical chip contact area to act as channels for the coolant, Gen II insert. Figure 2 shows the modification in the Gen II insert. The intention was to improve the coolant access to the proximity of the cutting edge where the highest temperature are generated during the machining process for HRSA materials. Based on the depth of cut of 1 mm, the channel features were placed at an arc angle of 30.2° on the rake face. In addition to the improved cooling, the channel features were also anticipated to influence the chip bending, thus lowering the chip contact area on the rake face in the secondary shear zone and lower the heat generated due to friction caused by mechanical interaction as seen in Figure 3c.

### 2.4. Coolant Interaction with Regular, Gen I and Gen II Inserts

High-pressure coolant on the rake and flank face creates a mechanical force by the hydro-wedge formation and thus, bending the chip see Figure 3a–c. Computational findings by Fang and Obikawa showed that textured surfaces created incomplete swirls and eddies. Those swirls led to a sharp increase of the heat transfer coefficient thus they significantly increased the cooling of the tool [12]. The intention of the surface features of Gen I and Gen II insert was therefore to make use of the presented theory of Fang and Obikawa and thereby promoting the circulation effect of the coolant, as illustrated in Figure 3d. Sustaining the coolant in the rake and flank contact regions should improve the heat transfer rate from the cutting zone. Thus, an increased tool life can be achieved.

## 3. Experimental

### 3.1. Workpiece Material—Alloy 718

The machining experiments were carried out on cast Alloy 718 with an average hardness of 381 ± 22 HV. In Table 3 nominal composition of the workpiece material is provided.

### 3.2. Machine Tool and High-Pressure Coolant Supply

**Figure 4:**
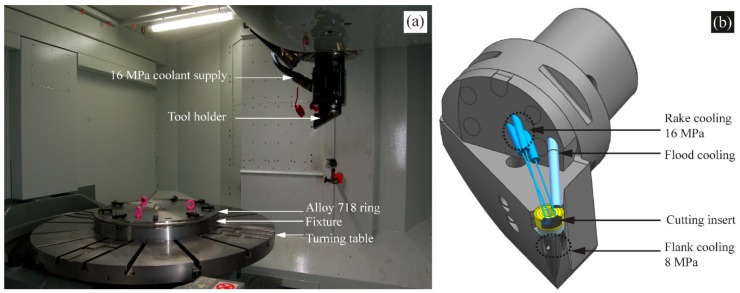
(**a**) 5-axis machine setup; (**b**) high-pressure coolant tool holder showing the rake and flank cooling.

A 5-axis CNC machine as shown in Figure 4a, was used to conduct the facing operations with high-pressure coolant on both rake and flank face. A specially designed tool holder (Figure 4b) was used for the precise focus of the coolant to the cutting edge. An emulsion concentration of 5% mixed with water was used as coolant. A coolant rake pressure of 16 MPa was used to supply coolant to the cutting edge through three nozzles (diameter of 0.8 mm) at approximately 11 L/min. For supply of coolant to the flank face, a pressure of 8 MPa and two nozzles (diameter of 1.2 mm) were used at approximately 12 L/min flow rate.

### 3.3. Spiral Cutting Length Calculation

**Figure 5:**
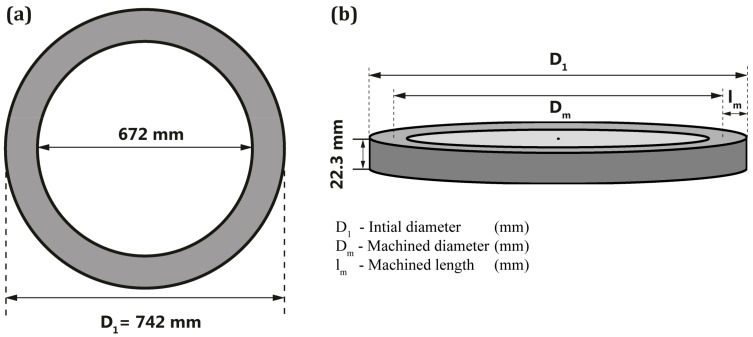
(**a**) Top view; and (**b**) side view of the machined ring with dimensions.

The dimensions of the ring used for the experiments are illustrated in Figure 5a,b. The machined length was approximately 25.1 mm. The spiral cutting length (SCL), for facing operation was calculated from the Equation (1), which is inversely related to the feed rate for constant length of cut.

(1) $$SCL=\left(\frac{D_{1}+D_{m}}{2}\right)\times \left(\frac{\pi }{1000}\right)\times \left(\frac{l_{m}}{f_{n}}\right)\;$$

### 3.4. Design of Experiments Based on Spiral Cutting Length and Cutting Speed

**Figure 6:**
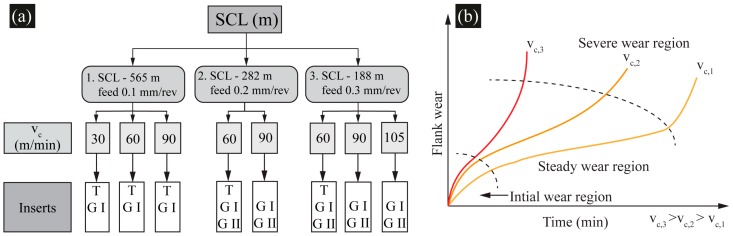
(**a**) Design of experiments (**b**) Flank wear progression with time for incremental cutting speeds [14].

The performance of the two generations of inserts (Gen I and Gen II) was compared with regular inserts (T) based on the tool wear investigation for three different SCL corresponding to three feed rates as given in Figure 6a. Depth of cut of 1 mm was kept constant for all the experiments. SCL of 565 m for *f_n_* 0.1 mm/rev was preferred for regular and Gen I inserts for low cutting speed and longer cutting time. The SCL of 282 m for *f_n_* 0.2 mm/rev and 188 m for 0.3 mm/rev were preferred for Gen II inserts. Since the increase in feed rates increases the tool-chip contact area on the rake face, interaction of the chip with the channel features on the rake will be promoted for higher feed rates. The cutting speed was chosen as a varying parameter since it is generally reported to have a direct correlation with the cutting temperature and the tool wear Figure 6b. Lower SCL’s were chosen, so inserts will have lower engagement time at higher cutting speed and feed rate. The flank pressure was kept zero for SCL 565 m, *v_c_* 30 m/min, *f_n_* 0.1 mm/rev—with regular and Gen I inserts—and 60 m/min, with the regular insert. For the rest of the cutting conditions, a rake pressure of 16 MPa and a flank pressure of 8 MPa were kept constant. The cutting conditions and inserts used for the different experiments are provided in Table 4.

## 4. Measurement Techniques

The tool wear was examined by combining light-optical microscopy (LOM), with 3-D measurements based on focus variation, scanning electron microscopy (SEM) and energy-dispersive X-ray spectroscopy (EDS).

- LOM was used to measure the flank wear and flank wear land profiles. Flank wear profiles were generated from 36 vertical measurements of the flank wear along the linear contact length of 3.6 mm. The individual vertical measurements were plotted into a graph.
- SEM was used to examine the tool wear at higher magnification on both rake and flank faces.
- EDS was used for elemental analysis of cutting tools after machining.
- Focus variation was used to investigate the area of the flank and rake wear, and for performing volume difference measurement.

### 4.1. Standard for Flank Wear and Notch Wear Measurements on Round Insert

**Figure 7:**
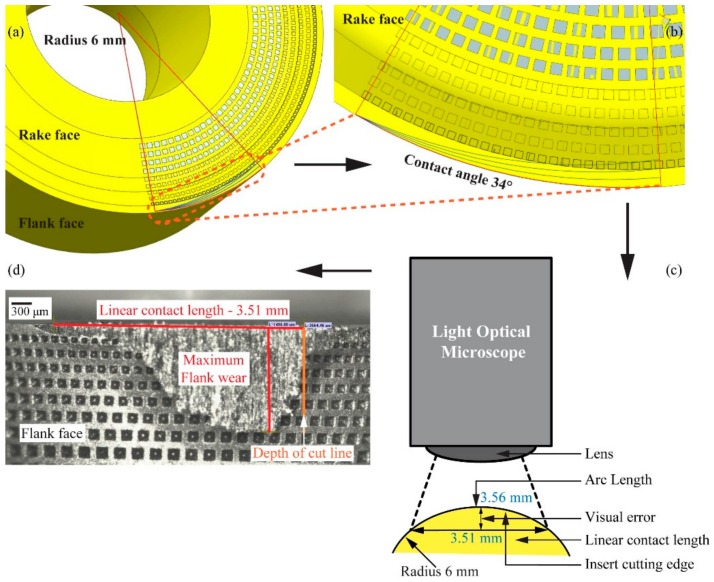
(**a**) Contact area of insert with *a_p_* of 1mm and for 6 mm insert radius; (**b**) magnified micrograph shows contact angle 34°; (**c**) conversation of arc length to liner contact length; and (**d**) illustration of maximum flank wear measurement in relation to linear contact length of 3.6 mm and depth of cut line [9,10].

The round geometry of the tool formed a necessity to set a standard to measure the flank wear. An axial depth of cut, *a_p_* of 1 mm would create a contact angle of 34° on the rake face of an insert with 6 mm in radius, Figure 7b. This then created an arc length of 3.56 mm and a linear contact length of 3.51 mm, Figure 7c. Considering the visual error the linear contact length was approximated to 3.6 mm for flank wear profile measurement and depth of cut notch wear, Figure 7d.

### 4.2. Standard for Tool-Chip Contact Area Measurements on Worn Round Insert

**Figure 8:**
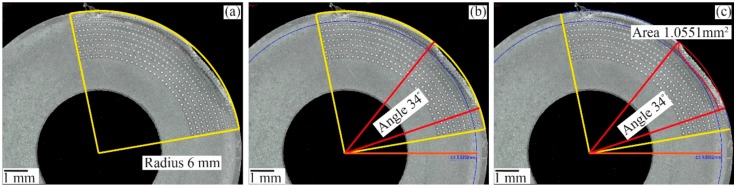
Illustration of (**a**) Three point arc to recreate the cutting edge (**b**) contact angle of 34° (**c**) tool-chip contact area measurement.

The tool-chip contact area of the round insert is measured based on the concept of contact angle and contact length. Firstly, a three-point arc was drawn to create the insert radius of 6 mm as can be seen in Figure 8a. This arc recreates the cutting edge as a reference for further measurements. Secondly, the contact angle of approximate 34 degrees measured in reference to worn surface as shown in Figure 8b. Thirdly, the tool-chip contact area is measured can be seen in Figure 8c.

## 5. Tool Wear Analysis

The tool wear investigation was categorized based on the spiral cutting length of 565, 282 and 188 m for different cutting speeds. Observed tool wear was reported in comparison between the different generations of inserts with regular insert for corresponding SCL. Followed by tool-chip contact area measurements for Gen I and Gen II insert.

### 5.1. Spiral Cutting Length 565 m and Feed Rate 0.1 mm/rev

#### 5.1.1. Flank and Rake Wear

**Figure 9:**
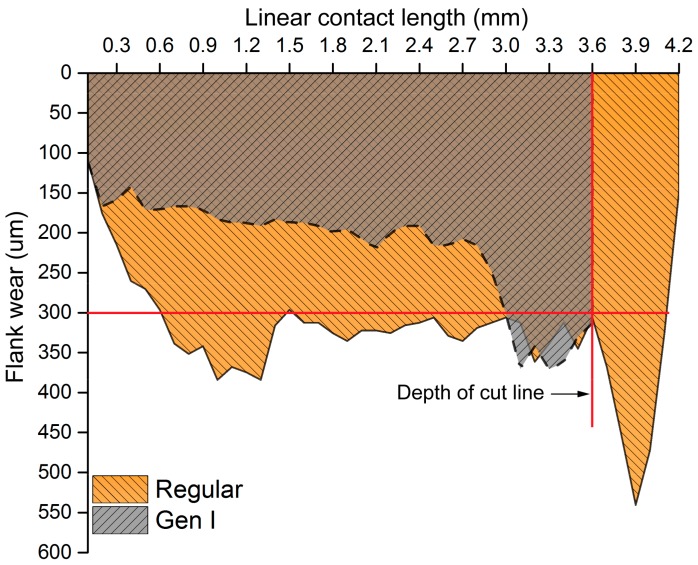
Flank wear profile of Regular and Gen I insert.

A lower cutting speed of 30 m/min was experimented for regular and Gen I insert without flank cooling. The aim was to investigate the effect of Gen I insert on flank wear and implicitly on heat dissipation. Figure 9 shows the flank wear profile of regular and Gen I inserts with industrial tool failure criteria of 300 µm given as a solid line. Gen I insert lowered the maximum flank wear approximately 46% compared to the regular insert. Significant depth of cut notch wear was observed on the regular insert beyond 3.6 mm of contact length.

**Figure 10:**
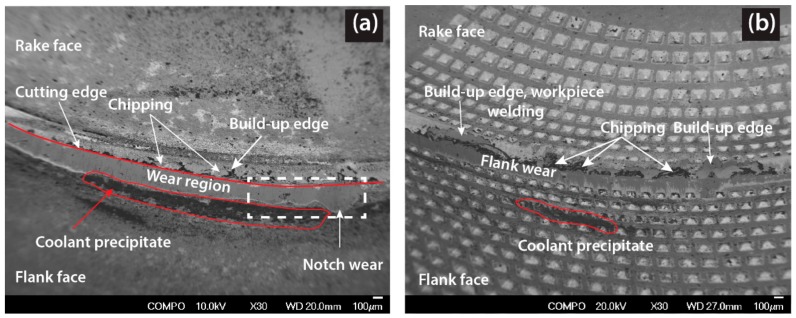
SEM micrograph comparison of tool wear for *v_c_* 30 m/min, *f_n_* 0.1 mm/rev, Rake Pressure (RP) 16 MPa and Flank Pressure (FP) 0 MPa, (**a**) Regular insert (**b**) Gen I insert.

Notch wear, as a groove formation similar to what is seen in the regular insert, was observed in the Gen I insert but within the depth of cut line, 3.6 mm, Figure 9. This can be attributed to the region of maximum chip thickness. Traces of build-up edges and adherence of workpiece material on the rake face were observed on the Gen I insert but only to a minor extent on the regular insert, see Figure 10. This observation can likely be related to the improved heat dissipation and cooling around the contact zone of Gen I insert. Despite the fact that there was no flank cooling, traces of dark regions were found below the flank wear as shown in Figure 10a. Elemental analysis determined the dark region to be calcium, which was from the coolant fluid used. Moreover, there were also traces of the workpiece material, i.e., nickel, chromium and iron observed in the flank wear region.

**Figure 11:**
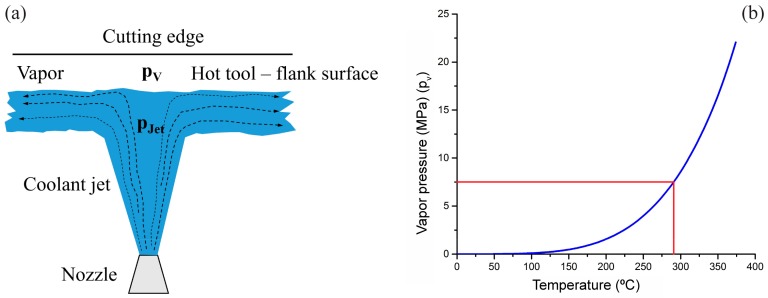
(**a**) Illustration of vapor barrier phenomenon occurring between hot tool flank surface and coolant (**b**) Vapor pressure of water as a function of temperature adopted from [17].

When the cutting tool temperature increases over the coolant boiling point, the so called Leidenfrost point, a vapor bar;rier film can be formed. This film acts as a barrier for the coolant to reach close to the proximity of the cutting edge, see Figure 11a [11,15,16]. When the jet pressure, *p_jet_*, is higher than the vapor pressure, *p_v_*, it can suppress/delay the formation of the vapor barrier by increasing the boiling point of the fluid [15]. This keeps the cutting temperature below the Leidenfrost point and facilitates the coolant’s access to the cutting edge. In Figure 11b, the vapor pressure is shown as a function of temperature, obtained from the table of thermodynamic properties of steam [17]. For instance, with the available flank pressure of maximum 8 MPa the corresponding temperature can be suppressed to a value between 280 °C and 290 °C. An increase in the cutting temperature would require a higher coolant pressure to avoid the formation of the Leidenfrost film [15]. Also, need to take into account the loss in pressure from the nozzle exit to the cutting edge.

**Figure 12:**
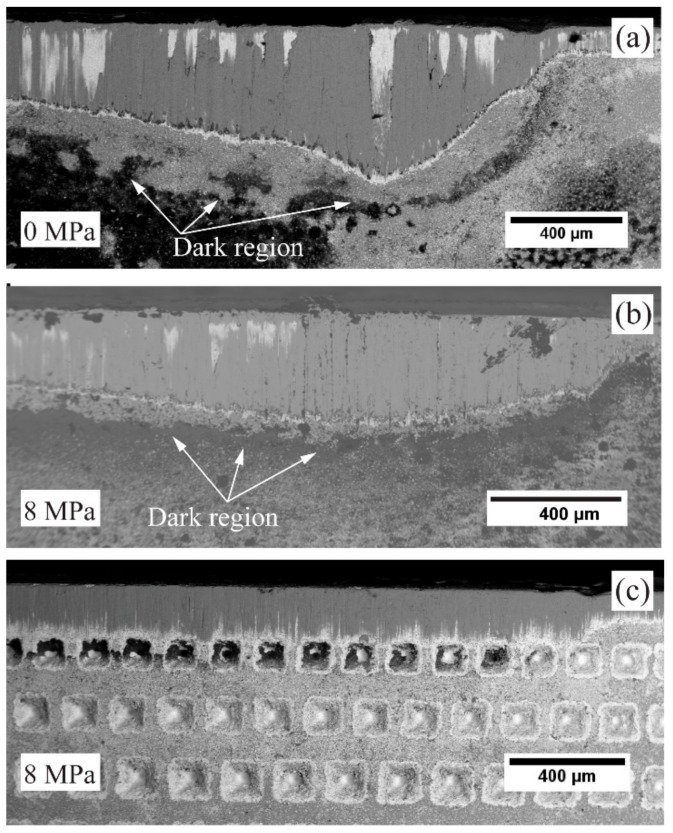
Flank wear comparison of regular insert (**a**) without flank cooling (0 MPa), (**b**) with 8 MPa flank cooling and (**c**) Gen I insert with 8 MPa flank cooling.

To investigate the Leidenfrost effect phenomenon and to understand the influence of flank coolant pressure on the tool wear, cutting speed was increased to 60 m/min when using the regular insert with and without flank pressure of 8 MPa. In Figure 12a,b, the corresponding SEM micrographs of the flank wear of the regular insert are shown.

The regular insert had a maximum flank wear of approx. 530 µm without flank cooling, while the flank wear was reduced to 306 µm with flank cooling [9]. Coolant boiling regions, which left precipitate from coolant below the flank wear seen as dark region from SEM micrograph, Figure 12a,b. It can be also be seen from the SEM micrographs dark region below the flank wear moved closer to the cutting edge when flank cooling was applied. The decrease in flank wear with high pressure coolant supply on the flank face was coherent with findings from Colak et al. [18]. Also, the Gen I insert was investigated with flank cooling of 8 MPa to understand tool wear as a direct correlation to the heat dissipation. The maximum flank wear was lowered to 200 µm with cooling compared to the regular insert, (306 µm). A substantially lower flank wear and a not as obvious dark region could be attributed to the improved heat dissipation in the Gen I insert in comparison to the regular insert.

#### 5.1.2. Elemental Analysis

**Figure 13:**
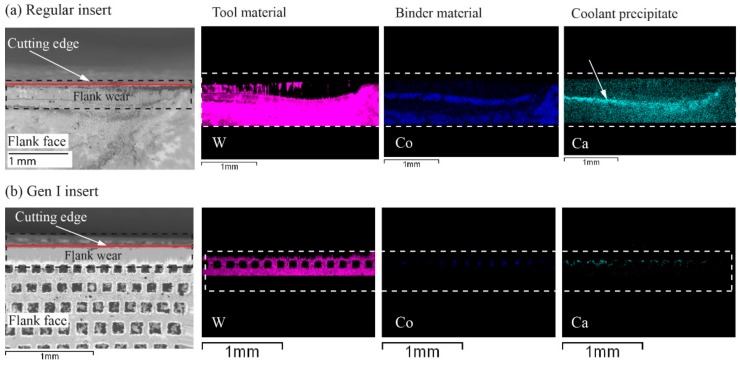
EDS analysis of flank wear for *v_c_* 60 m/min, *f_n_* 0.1 mm/rev, and flank pressure 8 MPa: (**a**) Regular insert; and (**b**) Gen I inserts.

EDS analysis on the regular and Gen I inserts with flank cooling revealed Ca precipitates on the flank faces. The regular insert had a clearly visible deposit as seen in Ca map in Figure 13a and can be correlated to the dark region seen in the SEM micrograph. The Ca deposit in relation to the dark region can be seen as traces of Leidenfrost film, which acts as a barrier. In contrast, the EDS results obtained on the Gen I insert for the same cutting conditions shows significantly less Ca as seen in Ca map in Figure 13b. This indicates that the thermal condition for machining with the Gen I insert was different and had an improved heat dissipation compared with the regular insert. In addition, it is likely that the coolant has a lower temperature than the Leidenfrost point when compared with the regular insert.

**Figure 14:**
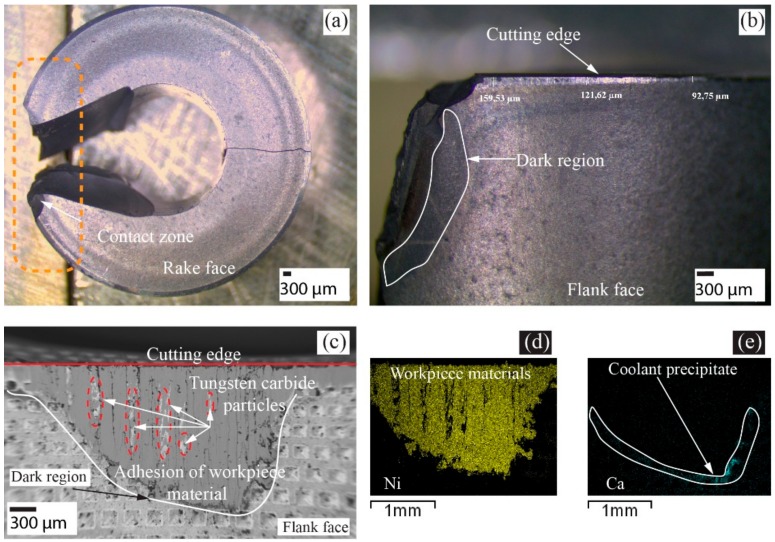
(**a**) Regular insert—catastrophic failure; (**b**) Regular insert—Dark region; (**c**) Flank wear of Gen I; (**d**) Elemental analysis of Nickel; (**e**) Elemental analysis of Calcium.

The cutting speed was increased to 90 m/min, and the regular and Gen I inserts were investigated. As shown in Figure 14a,b, the regular insert had a catastrophic failure and showed traces of dark region along the tool wear. As a result, further investigations at higher cutting speeds were not conducted. The Gen I insert had an extensive flank wear of 1.4 mm without tool breakage, as shown in Figure 14c. EDS analysis of the flank wear region as shown in Figure 14d,e showed the deposition of nickel on the flank wear region and calcium precipitation below the flank wear. Improved tool life of Gen I and Ca precipitate can be related to the improved heat dissipation from the cutting zone that led the Gen I insert to operate at higher cutting speed compared to regular insert.

### 5.2. Spiral Cutting Length 282 m and Feed Rate 0.2 mm/rev

**Figure 15:**
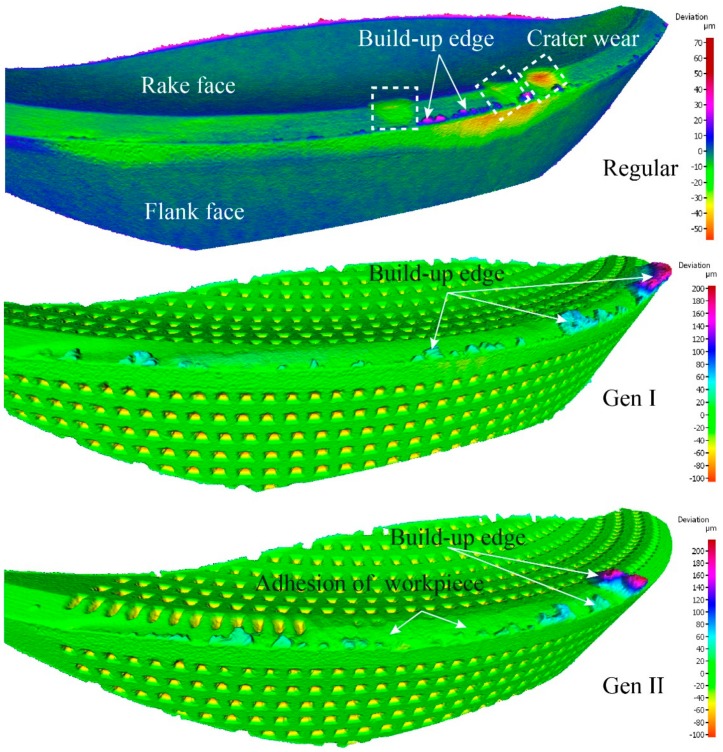
Illustration of observed different tool wear mechanism from volume difference measurement of Regular, Gen I and Gen II inserts. The topography scans are presented as the geometrical volume deviation from an unworn inserts.

For a cutting speed of 60 m/min, and feed rate of 0.2 mm/rev the maximum flank wear was observed in the regular insert, whereas the Gen II insert had a reduced flank wear by 44%. Volumetric analysis of three inserts revealed the existence of crater wear on the regular insert whereas in Gen I and Gen II inserts, respectively, there was no crater wear observed, Figure 15. 

Kalpakjian [19], stated “the location of maximum crater depth coincides with the location of the maximum temperature at the tool-chip interface”. Volume removed from the regular insert rake face was concentrated in the crater area. In case of Gen I and Gen II inserts, respectively, the volume of material adhered to the rake face was approximately twice as much as compared to the regular insert. The presence of the build-up edge and deposits of workpiece material on the inserts indicates the inserts machined at low cutting speed. Absence of crater wear and adherence of workpiece material on Gen I and Gen II presents the improved cooling of inserts compared to the regular insert.

**Figure 16:**
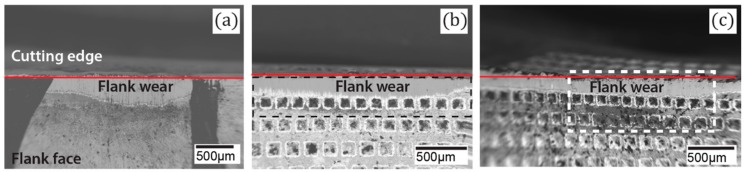
SEM images of insert flank face in the region of flank wear (**a**) Regular, (**b**) Gen I and (**c**) Gen II.

SEM micrographs of Regular, Gen I and Gen II inserts, respectively, are shown in Figure 16a–c. Below the flank wear of the regular insert a strong layer of calcium traces was observed [11]. In the Gen I insert, the Ca-layer was closer to the cutting edge, while the calcium precipitates distributed evenly on the flank face of Gen II insert [11]. The wide area of calcium deposit indicates that there is no strict boundary created by the possible Leidenfrost film. This could be related to the improved tool life of the Gen II insert compared to Gen I and regular insert.

When the cutting speed was further increased to 90 m/min, the Gen II insert had a 28% reduced flank wear compared to the Gen I insert [10]. Both the Gen I and Gen II inserts had extensive flank wear of more than 1 mm. However, no catastrophic failure was observed. 

### 5.3. Spiral Cutting Length 188 m and Feed Rate 0.3 mm/rev

#### 5.3.1. Volumetric Analysis

**Figure 17:**
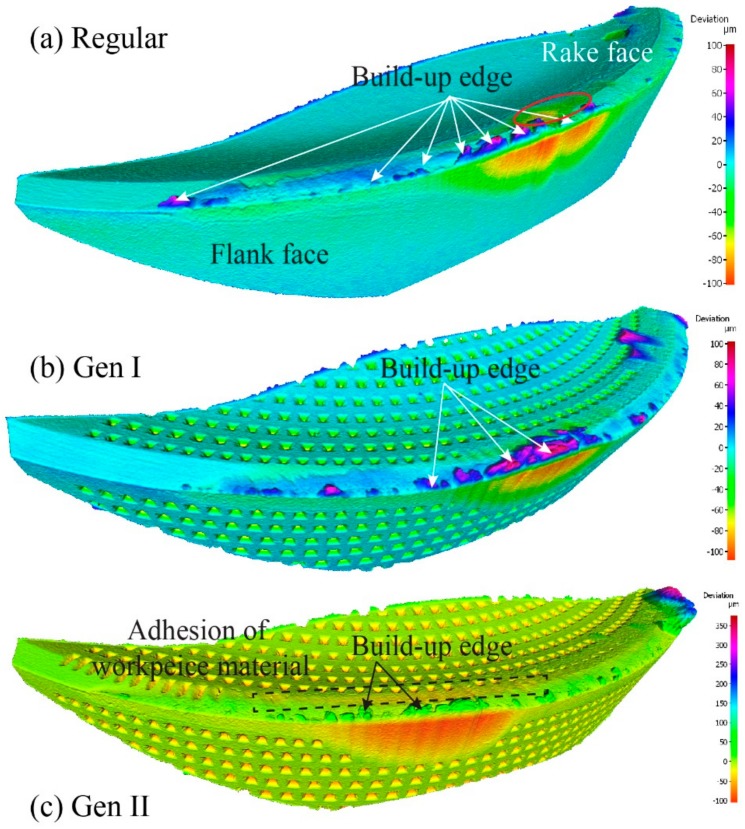
Volumetric analysis result at the inserts from top (**a**) Regular, (**b**) Gen I, and (**c**) Gen II. The topography scans are presented as the geometrical volume deviation from an unworn inserts.

For the cutting speed of 60 m/min the regular, Gen I and Gen II inserts were investigated. Results from the flank wear analysis were ambiguous [9,10]. To clarify, the volumetric analysis was performed on the cutting tools seen in Figure 17. Positive values in the scale represents the volume added and negative values illustrate the volume removed from the cutting tool by wear.

Tool wear observations showed similar cutting edge deformation, adhesion of workpiece material and build-up edge at a different level. Surprisingly, the Gen II insert had the maximum flank wear compared to the other inserts. Therefore, further cutting experiments and investigations are needed for this SCL and cutting condition in order to understand the tool wear mechanism.

#### 5.3.2. Flank Wear Profile Measurement and Flank Wear Area Calculations

**Figure 18:**
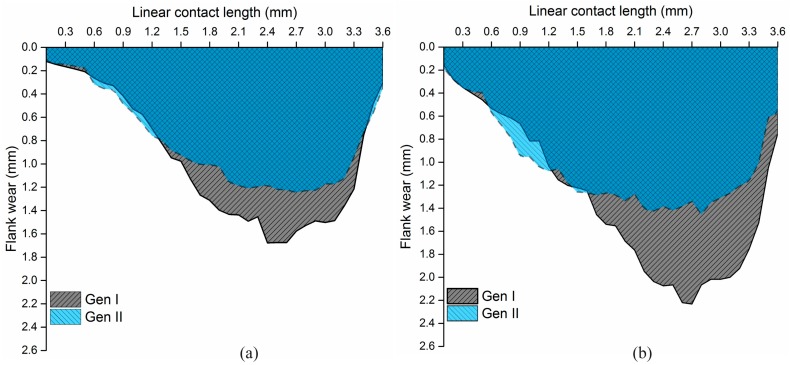
Flank wear profile of Gen I and Gen II inserts for *f_n_* 0.3 mm/rev (**a**) *v_c_* 90 m/min (**b**) *v_c_* 105 m/min.

Cutting speeds were further increased to 90 and 105 m/min, respectively, and Gen I and Gen II inserts were investigated. Both the inserts were used to machine Alloy 718 without any signs of tool breakage. The flank wear profiles for *v_c_* 90 m/min is shown in Figure 18a, and for *v_c_* 105 m/min is shown in Figure 18b, respectively. Gen I and Gen II inserts had an extensive flank wear, however, Gen II insert for cutting speeds 90 and 105 m/min showed a reduced flank wear of approx. 24% and 35%, respectively. Interestingly, flank wear of the Gen II insert for the cutting speed of 90 and 105 m/min was approximately the same.

**Figure 19:**
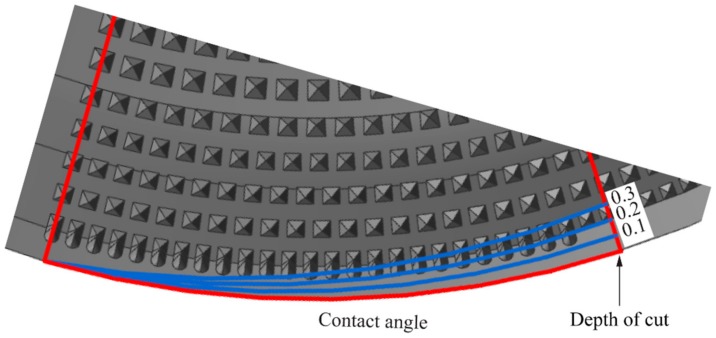
Theoretical chip contact area on Gen II insert based on different feed rates.

In addition, an interesting observation was the shape of the flank wear profiles as shown in Figure 18. The flank wear profile of the Gen I insert was peak shaped, while the flank wear profile was more of a bell shaped for the Gen II insert [10]. The profiles were consistent for all cutting speeds and SCL’s for Gen I and Gen II inserts. An increase in feed rate leads to an increased theoretical tool-chip contact area on the rake face, as shown in Figure 19. The maximum flank wear region in the middle of the contact length can be attributed to the two-flank nozzle position, located at the beginning and end of the contact length.

Calculation of flank wear area were performed using 3-D measurement tools and are tabulated in Table 5. The results show that the worn area of Gen II inserts was reduced by 13–18% compared to Gen I insert for the corresponding cutting conditions.

### 5.4. Tool-Chip Contact Area

The Gen II insert with channel design on the rake face was designed with the intention to improve coolant accessibility to the proximity of the cutting edge, where more heat is generated. It also creates a possibility for the coolant to penetrate beneath the chip, which leads to lift of the chip on the rake face. Thus, the channel design is lowering the tool-chip contact length, and the heat generated due to mechanical friction in the secondary shear zone. The theoretical chip contact area for feed rate of 0.1, 0.2 and 0.3 mm/rev on the rake face in correlation with the channel design is illustrated in Figure 19.

**Figure 20:**
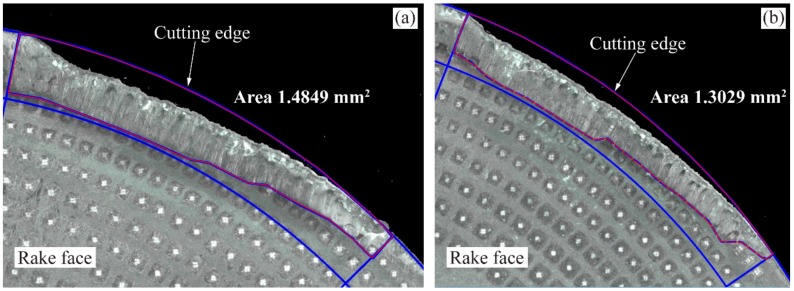
Tool-chip contact area of the rake face of the inserts for *v_c_* 90 m/min and *f_n_* 0.3 mm/rev, (**a**) Gen I and (**b**) Gen II.

Gen I and Gen II inserts were investigated on the rake face for SCL of 282 m and 188 m, respectively, where both the cutting inserts had the cutting edge deterioration at varying levels. Tool-chip contact area on the rake face were calculated from a 3-D measurement tool and results are tabulated in Table 6. For a feed of 0.3 mm /rev and a cutting speed of 90 m/min, the contact areas of Gen I and Gen II inserts are shown in Figure 20. Overall results showed that the Gen II insert has decreased the tool-chip contact area between 12–17% in comparison to the Gen I insert.

**Figure 21:**
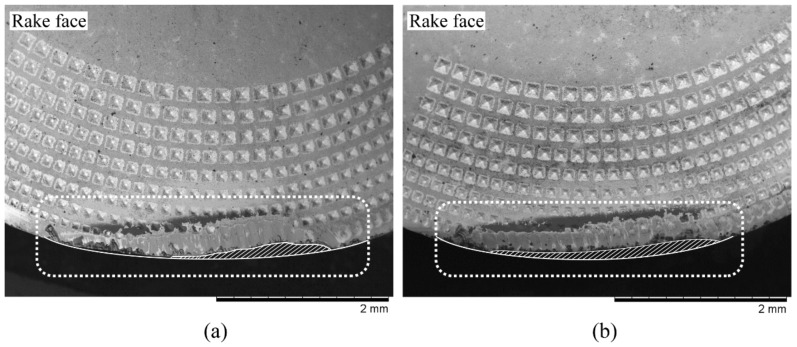
Comparison of cutting edge deterioration on the rake face for *v_c_* 90 m/min, *f_n_* 0.2 mm/rev, (**a**) Gen I (**b**) Gen II.

The cutting edge deterioration and the contact area for Gen I and Gen II insert are shown in Figure 20 and Figure 21 and compared with the theoretical chip contact areas (see Figure 19). Gen II insert had less cutting edge deterioration and follows to a greater extent the shape of the theoretical chip contact area as the wear progressed. For the case of Gen I insert, cutting edge deterioration did not follow the theoretical chip contact area. Higher wear on the cutting edge was localized with respect to area of maximum chip thickness.

## 6. Conclusions

Results on Gen I and Gen II inserts regarding tool wear in relation to heat dissipation showed that both insert designs resulted in better cooling and led to improvements in tool life compared to the regular insert. Gen I and Gen II insert had the same type of tool wear mechanism but at a different level. Gen II insert showed improvement in the tool life, a reduced flank wear by 20–35%, tool-chip contact and flank wear area by 12–17% compared to Gen I insert. In addition, chemical analysis of the dark region below the flank wear of the inserts revealed the presence of calcium precipitates as a residue from the coolant. Calcium precipitate deposits were closer to the cutting edge for Gen I and Gen II inserts, which can be attributed to the better cooling of the inserts led to the improved access of the coolant closer to the cutting edge.

## Figures and Tables

**Table 1:** Insert nomenclatures.

Inserts	Regular/T Gen I/G I Gen II/G II
Insert thickness (mm)	4.76
Inscribed circle diameter (mm)	12
Face land width (mm)	0.2
Rake angle (°)	0
Clearance angle (°)	7
Face land angle (°)	15
Type of insert	Uncoated carbide insert
Insert shape	Round
Chip breaker	No

**Table 2:** Surface area calculations for different geometries for constant volume.

S.no	Geometry	Input (mm)	Total Surface Area (T.S.A) (mm^2^)	Results (mm^2^) (TSA-BSA)
1	Square pyramid	*a* = 4; *h* = 9	$A=a^{2}+2a\sqrt{\frac{a^{2}}{4}+h^{2}}$	89.76 − 16 = 73.76
2	Cone	*r* = 2.2567; *h* = 9	$A=\pi r\left(r+\sqrt{h^{2}+r^{2}}\right)$	81.78 − 16 = 65.78
3	Cylinder	*r* = 2.2567; *h* = 3	$A=2\pi rh+2\pi r^{2}$	74.54 − 16 = 58.54

**Table 3:** Nominal chemical composition (wt %) of Alloy 718 [13].

Elements	Ni	Cr	Nb	Mo	Ti	Al	Co.	Si	Mn-Cu	C	Fe
**wt %**	53.4	18.8	5.27	2.99	1.02	0.50	0.17	0.12	0.07	0.03	Bal

**Table 4:** Cutting parameters for facing operation of Alloy 718.

SCL (m)	*a_p_* (mm)	*v_c_* (m/min)	*f_n_* (mm/rev)	Pressure (MPa)		Tests Conducted		
				Rake	Flank	Regular	Gen I	Gen II
565	1	30	0.1	16	0	X	X	-
565	1	60	0.1	16	0	X	-	-
565	1	60	0.1	16	8	X	X	-
565	1	90	0.1	16	8	X	X	-
282	1	60	0.2	16	8	X	X	X
282	1	90	0.2	16	8	-	X	X
188	1	60	0.3	16	8	X	X	X
188	1	90	0.3	16	8	-	X	X
188	1	105	0.3	16	8	-	X	X

- No tests were conducted.

**Table 5:** Results of flank wear area calculation.

S.no	SCL (m)	Cutting Speed (m/min)	Feed Rate (mm/rev)	Gen I (mm^2^)	Gen II (mm^2^)	Difference in Percentage (%)
**1**	282	90	0.2	3.8161	3.1380	17.8
**2**	188	90	0.3	3.5583	2.9307	17.6
**3**	188	105	0.3	4.4782	3.9027	12.9

**Table 6:** Calculation of chip contact area [mm^2^].

S.no	SCL (m)	Cutting Speed (m/min)	Feed Rate (mm/rev)	Gen I (mm^2^)	Gen II (mm^2^)	Difference in Percentage (%)
1	282	90	0.2	1.2831	1.0551	17.7
2	188	90	0.3	1.4849	1.3029	12.3
3	188	105	0.3	1.6165	1.4232	12

## References

[1] Rahim E., Warap N., Mohid Z., Aliofkhazraei M. (2015). Thermal-assisted machining of nickel-based alloy. Superalloys.

[2] Pigott R.J.S., Colwell A.T. (1952). Hi-jet system for increasing tool life. SAE Tech. Pap..

[3] Courbon C., Kramar D., Krajnik P., Pusavec F., Rech J., Kopac J. (2009). Investigation of machining performance in high-pressure jet assisted turning of Inconel 718: An experimental study. Int. J. Mach. Tools Manuf..

[4] Krämer A., Klocke F., Sangermann H., Lung D. (2014). Influence of the lubricoolant strategy on thermo-mechanical tool load. CIRP J. Manuf. Sci. Technol..

[5] Courbon C., Sajn V., Kramar D., Rech J., Kosel F., Kopac J. (2011). Investigation of machining performance in high pressure jet assisted turning of Inconel 718: A numerical model. J. Mater. Process. Technol..

[6] Pettersson U., Jacobson S. (2003). Influence of surface texture on boundary lubricated sliding contacts. Tribol. Int..

[7] Kawasegi N., Sugimori H., Morimoto H., Morita N., Hori I. (2009). Development of cutting tools with microscale and nanoscale textures to improve frictional behavior. Precis. Eng..

[8] Lei S., Devarajan S., Chang Z. (2009). A study of micropool lubricated cutting tool in machining of mild steel. J. Mater. Process. Technol..

[9] Tamil Alagan N., Beno T., Wretland A. (2016). Next Generation Insert for Forced Coolant Application in Machining of Inconel 718. Mater. Sci. Forum.

[10] Tamil Alagan N., Beno T., Wretland A. (2016). Investigation of Modified Cutting Insert with Forced Coolant Application in Machining of Alloy 718. Procedia CIRP.

[11] Jäger H., Alagan N.T., Holmberg J., Beno T., Wretland A. (2016). EDS Analysis of Flank Wear and Surface Integrity in Machining of Alloy 718 with Forced Coolant Application. Procedia CIRP.

[12] Fang Z., Obikawa T. (2017). Cooling performance of micro-texture at the tool flank face under high pressure jet coolant assistance. Precis. Eng..

[13] Rahman M., Seah W.K.H., Teo T.T. (1997). The machinability of inconel 718. J. Mater. Process. Technol..

[14] Trent E.M. (2000). Metal Cutting.

[15] Sørby K., Tønnessen K. (2006). High-pressure cooling of face-grooving operations in Ti6Al4V. Proc. Inst. Mech. Eng. Part B J. Eng. Manuf..

[16] Van Limbeek M.A.J., Shirota M., Sleutel P., Sun C., Prosperetti A., Lohse D. (2016). Vapour cooling of poorly conducting hot substrates increases the dynamic Leidenfrost temperature. Int. J. Heat Mass Transf..

[17] Straub J. (1985). NBS/NRC steam tables. VonL. Haar, J. S. Gallagher undG. S. Kell. Hemisphere Publishing Corp., Washington-New York-London 1984. 1. Aufl., XII, 320 S., geb., $ 34.50. Chem. Ing. Tech..

[18] Çolak O. (2012). Investigation on machining performance of Inconel 718 under high pressure cooling conditions. Strojniski Vestnik J. Mech. Eng..

[19] Kalpakjian S., Schmid S.R., Schmid S.R., Vijay Sekar K.S. (2010). Manufacturing Engineering and Technology.

